# Preventing Post-Transfusion Hepatitis by screening blood donors for IgM Antibody to Hepatitis B core antigen

**DOI:** 10.4103/0974-777X.68526

**Published:** 2010

**Authors:** Nilima G Sawke, GK Sawke

**Affiliations:** *Department of Pathology, People’s College of Medical Sciences & Research Centre, Bhopal, Madhya Pradesh, India*

Transfusion-associated hepatitis B viral infection (TAHBV) continues to be a major problem in India even after adoption of mandatory screening for HBsAg (hepatitis B surface antigen) detection by ELISA (enzyme-linked immunosorbent assay) method. The high incidence of TAHBV is reported in patients receiving multiple transfusions. It has been demonstrated that some HBsAg-negative and anti HBc antigen positive (anti-hepatitis B core antigen positive) individuals continue to replicate hepatitis B virus (HBV). The negative HBsAg in the blood of apparently healthy individuals may not be sufficient to ensure absence of circulating HBV. Thus the blood containing anti-HBc antigen with or without detectable presence of HBsAg might be infectious.

As of today, the blood banks of many countries have adopted anti-HBc testing to decrease HBV transfusion risk, while others have not. Anti-HBc testing is not mandatory in blood banks of many developing countries, and only HBsAg testing by ELISA is used as screening test for HBV infection.

The prevalence rate of HBsAg-positive adult, healthy donor population in the country is around 4.7%. Data suggests that in the general population about 75% carriers acquire infection by horizontal spread during early childhood, and the remaining acquire it by vertical (mother-to-child) transmission.[1] A study by Patwari *et al*. reported higher incidence of HBV-carrier state in transfusion recipients as compared to the general population (12.3% *vs*. 3.6%). These authors found the incidence of TAH-B (transfusion associated hepatitis B) to be 11%.[2] Despite adequate screening of blood, multiple-transfused cardiac surgery patients, hemodialysis patients, thalassemics and hemophiliacs showed higher incidence of HBV infection.

HBV consists of several proteins or antigens to which antibodies are formed. A surface antigen protein HBsAg is present on the outer envelope of the virus, and it can be found floating free in the plasma. Antibodies are also produced against the two proteins present within the core, hepatitis B core antigen (HBcAg) and hepatitis Be antigen (HBeAg).

HBV DNA is the first marker to be detected by polymerase chain reaction (PCR) before HBsAg reaches detectable levels. But the PCR or nucleic acid amplification test (NAT) is not performed routinely in blood centers. HBcAg is present in the serum but is undetectable; however, IgM anti-HBc is the first antibody to appear and persists for about six months. IgG anti-HBc is found lifelong in persons who have been infected with HBV and is a marker of past or current viral replication.[3] In the following conditions, individuals who tested HBsAg negative may have undetectable circulating viruses present in the blood:

1. Carriers with HBsAg below the detection level can transmit HBV by blood transfusion.
2. Subjects infected with HBV may show HBsAg negative result owing to point mutation in the pre-core region of the virus, resulting in inability to synthesize HBsAg. Fulminant hepatitis developed in recipients of HBsAg-negative blood from such donors infected with mutated virus. In all those donors, high levels of anti-HBc were present.
3. In acute infection, there are two periods when HBsAg may be undetectable although the subject can transmit HBV — during early incubation period when both HBsAg and anti-HBc are undetectable; and after clearance of HBsAg but before anti-HBs has become detectable (diagnostic window). In this phase, anti-HBc and anti-HBe can be detected.

To encourage voluntary blood donation should be the first step of prevention. In Japan a change from paid donation to voluntary donation decreased the incidence of TAH(transfusion associated hepatitis) from 51% to 16%. Second step is to use sensitive tests for detection of HBV. Anti-HBc testing was introduced in the United States as a surrogate marker to identify units of donated blood that posed increase risk of transmission of HBV infection.[4,5]

In India, the reported incidence of anti-HBc among blood donors ranges from 17% to 29%.[6] Most of the studies done for estimation of anti-HBc among blood donors, have used kits for total anti-HBc (both IgG and IgM). The anti-HBc IgG may be found positive in an affected individual who has had past infection of HBV, even in presence of protective levels of anti-HBs antibodies, and therefore may not be infective. But the anti-HBc IgM is a marker of recent infection and so considered to be a more specific marker for HBV infection during the window period. Studies have shown that all blood units positive for anti-HBc may not be infectious, especially if the donor sera have adequate titer of anti-HBs.[7]

In India seroprevalence of anti-HBc is quite high as compared to western countries; hence screening of donor blood for total anti-HBc may not be practically important and cannot be the criterion to discard blood units, whereas the anti-HBc IgM–reactive samples with negative HBsAg test may identify the potentially infectious blood units.

Retrospective studies on regular blood donors and their respective recipients are essential to know the rate of HBV transmissions via anti-HBc–positive and HBsAg-negative donations. Routine anti-HBc IgM screening of blood donors could probably prevent some transfusion-transmitted HBV infections.

To reduce the risk of transfusion-associated hepatitis B, test for anti-HBc IgM may be included in routine screening of donors’ blood as it has been proved to be an excellent indicator of occult HBV during window period. However, awareness and education of donors regarding the modes of HBV transmission, a stringent one-to-one donor screening and increasing the voluntary donor base should also be implemented to minimize the rate of transfusion-associated hepatitis B.

## References

[1] Tandon BN, Gandhi BM, Joshi YK (1984). Etiological spectrum of viral hepatitis and prevalence of markers of hepatitis A and B virus infection in North India. Bull World Health Organ.

[2] Patwari SI, Irshad M, Gandhi BM, Joshi YK, Nundy S, Tandon BN (1986). Post transfusion hepatitis: A prospective study. Indian J Med Res.

[3] Williams EF, Janeau PC, Zitzmann MB, Harmening DM (2005). Modern blood banking and transfusion practices.

[4] Kojima M, Shimzu M, Tsuchimochi T (1991). Posttransfusion fulminant hepatitis B associated with pre-core defective HBV mutants. Vox Sang.

[5] Kapoor D, Sarin SK (1997). Transfusion associated hepatitis: Indian scenario. Indian Pediatr.

[6] Kant L, Arora NK, Sarin SK, Singhal AK (1996). Transmission of hepatitis B virus in children: Indian scenario. Hepatitis B in India: Problems and prevention.

[7] Molijn MH, van der Linden JM, Ko LK, Gorgels J, Hop W, van Rhenen DJ (1997). Risk factors and anti-HBc reactivity among first time blood donors. Vox Sang.

